# A putative scenario of how *de novo* protein-coding genes originate in the *Saccharomyces cerevisiae* lineage

**DOI:** 10.1186/s12864-024-10669-5

**Published:** 2024-09-05

**Authors:** Tetsushi Yada, Takeaki Taniguchi

**Affiliations:** 1 Department of Bioscience and Bioinformatics, Kyushu Institute of Technology, Fukuoka, Japan; 2 Mitsubishi Research Institute, Inc., Tokyo, Japan

**Keywords:** *de novo* gene birth, Sequence analysis, *Saccharomyces cerevisiae*

## Abstract

**Background:**

Novel protein-coding genes were considered to be born by re-organization of pre-existing genes, such as gene duplication and gene fusion. However, recent progress of genome research revealed that more protein-coding genes than expected were born *de novo*, that is, gene origination by accumulating mutations in non-genic DNA sequences. Nonetheless, the in-depth process (scenario) for *de novo* origination is not well understood.

**Results:**

We have conceived bioinformatic analysis for sketching a scenario for *de novo* origination of protein-coding genes. For each *de novo* protein-coding gene, we firstly identified an edge of a given phylogenetic tree where the gene was born based on parsimony. Then, from a multiple sequence alignment of the *de novo* gene and its orthologous regions, we constructed ancestral DNA sequences of the gene corresponding to both end nodes of the edge. We finally revealed statistical features observed in evolution between the two ancestral sequences. In the analysis of the *Saccharomyces cerevisiae* lineage, we have successfully sketched a putative scenario for *de novo* origination of protein-coding genes. (1) In the beginning was GC-rich genome regions. (2) Neutral mutations were accumulated in the regions. (3) ORFs were extended/combined, and then (4) translation signature (Kozak consensus sequence) was recruited. Interestingly, as the scenario progresses from (2) to (4), the specificity of mutations increases.

**Conclusion:**

To the best of our knowledge, this is the first report outlining a scenario of *de novo* origination of protein-coding genes. Our bioinformatic analysis can capture events that occur during a short evolutionary time by directly observing the evolution of the ancestral sequences from non-genic to genic. This property is suitable for the analysis of fast evolving *de novo* genes.

## Introduction

Almost all protein-coding genes were born by re-organization of pre-existing genes, such as gene duplication and gene fusion, and it has been considered that few of them were born *de novo*, that is, gene origination by accumulating mutations in non-genic DNA sequences [1]. However, it has become clear that more protein-coding genes than expected were born *de novo*, as comprehensive data, such as RNA-seq and ribosome profiling, have been released [2–7]. Moreover, their biological functions have been identified in some cases [8]. Nonetheless, the in-depth process (scenario) for the *de novo* origination is not well understood.

A comprehensive catalogue of *de novo* protein-coding genes in the *Saccharomyces cerevisiae* genome has been compiled by Carvunis et al. [2]. The catalogue consists of 777 annotated and 1,139 unannotated ORFs. The 777 annotated ORFs were identified based on homology search against protein and nucleotide sequences of the 14 other yeast species up to *Schizosaccharomyces pombe*. According to their conservation levels over the Ascomycota phylogeny, they were classified into four groups S${}_1$ ∼ S${}_4$, that is, ORF${}_{\textrm{S}_1}$ is specific to *S. cerevisiae*, while ORF${}_{\textrm{S}_2}$, ORF${}_{\textrm{S}_3}$ and ORF${}_{\textrm{S}_4}$ are conserved from *S. cerevisiae* to *Saccharomyces paradoxus*, *Saccharomyces mikatae* and *Saccharomyces bayanus*, respectively. The 1,139 unannotated ORFs, which is denoted by ORF${}_{\textrm{S}_0}^+$, were identified based on transcription and translation evidence, that is, RNA-seq and ribosome profiling data in rich and starvation conditions. They are unannotated ORFs specific to *S. cerevisiae*, longer than 30 nucleotides and free from overlap with annotated ORFs on the same strand. Note that ORF${}_{\textrm{S}_0}^+$s would exhibit features intermediate between genes and non-genes. A considerable number of them might lose the ability to be transcribed and translated. However, it is notable that *de novo* protein-coding genes are expected to gradually emerge from them.

## Approach

Inspired by the above data, we have conceived bioinformatic analysis for sketching a scenario for *de novo* origination of protein-coding genes. Our analysis can be applied when the following conditions are met: (a) a number of *de novo* protein-coding genes in a species are catalogued, (b) their conservation levels over phylogeny are identified, (c) a phylogenetic tree of the closely relating species is given, and (d) their genome sequences are available. The data above meets the conditions.

**Fig. 1:**
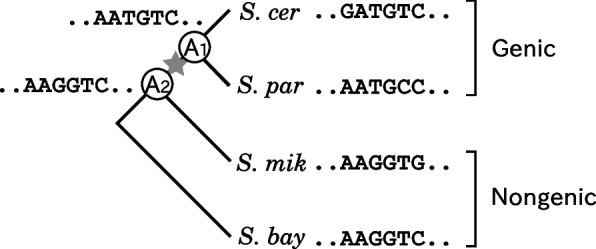
Schematic diagram of our bioinformatic analysis. This shows the analysis of a *de novo* protein-coding gene ORF${}_{\textrm{S}_2}$ of *S. cerevisiae*, which is conserved in *S. cerevisiae* and *S. paradoxus*. According to parsimony, we can consider that the gene was born on the edge (★) of a given phylogenetic tree. Then, we can construct the most likely ancestral DNA sequences of the gene corresponding to both end nodes (A${}_1$ and A${}_2$) of the edge from a multiple sequence alignment of the gene and its orthologous regions. Note that an ancestral sequence of A${}_1$ is genic, while that of A${}_2$ is non-genic. We then reveal statistical features observed in evolution from the ancestral sequence of A${}_2$ to that of A${}_1$, and finally sketched a putative scenario for *de novo* origination of protein-coding genes based on the statistical features observed

Figure 1 shows the schematic diagram of our bioinformatic analysis. For each *de novo* protein-coding gene catalogued, we firstly identified an edge of a given phylogenetic tree where the gene was born based on parsimony. We secondly constructed the most likely ancestral DNA sequences of the gene corresponding to both end nodes of the edge from a multiple sequence alignment of the *de novo* gene and its orthologous regions. Note that ancestral sequences of the both parent and child nodes are non-genic and genic, respectively. We thirdly revealed statistical features observed in evolution from the parent ancestral to the child ancestral sequences. Our bioinformatic analysis enables us to capture statistical features of DNA sequences observed in evolution from non-genic to genic sequences. Based on the statistical features observed, we finally sketched a putative scenario for *de novo* origination of protein-coding genes.

## Methods

### Data

We list below a set of data which we used for the bioinformatic analysis. Genome sequences of *Saccharomyces sensu stricto* species, that is, *S. cerevisiae*, *S. paradoxus*, *S. mikatae* and *S. bayanus*, were obtained from Saccharomyces Genome Resequencing Project (SGRP) and Saccharomyces Genome Database (SGD). See Table 1 for the detailed information of the data. A well-studied phylogenetic tree of *S. sensu stricto* species was given by Kellis et al. [9]. A comprehensive catalogue of *de novo* protein-coding genes of *S. cerevisiae* was compiled by Carvunis et al. [2]. In this catalogue, genomic locations of *de novo* protein-coding genes and their conservation levels over the phylogeny are shown. The detailed contents of the catalogue are described in the last paragraph of ‘Introduction’.

**Table 1:** Genome sequence data. A set of genome sequence data which was used for our bioinformatic analysis is listed

Species	*S. cerevisiae*
URL File	ftp.sanger.ac.uk/pub/users/dmc/yeast/genomes/cere.fa
Date	10-Oct-2007
Species	*S. paradoxus*
URL File	http://downloads.yeastgenome.org/sequence/fungi/S_paradoxus/NRRL-Y17217/NRRL-Y17217_MIT_2003_AABY01000000.fsa.gz
Date	28-Mar-2003
Species	*S. mikatae*
URL File	http://downloads.yeastgenome.org/sequence/fungi/S_mikatae/IFO1815/IFO1815_MIT_2003_AABZ01000000.fsa.gz
Date	28-Mar-2003
Species	*S. bayanus*
URL File	http://downloads.yeastgenome.org/sequence/fungi/S_bayanus/623-6C/623-6C_WashU_2005_AACG02000000.fsa.gz
Date	29-Aug-2005

### Bioinformatic analysis

According to genomic locations of *de novo* protein-coding genes shown in the catalogue, we extracted their nucleotide sequences from the *S. cerevisiae* genome. Each extracted sequence includes flanking regions which are 500 nt sequence long from both ends of a gene. Since statistics calculated from short *de novo* protein-coding genes are unreliable, we discarded ones whose ORF lengths are < 60 nt. The number of *de novo* protein-coding genes discarded was 692, which consist of 691 ORF${}_{\textrm{S}_0}^+$s and 1 ORF${}_{\textrm{S}_1}$. In addition, to calculate background statistics, we randomly extracted 6,006 intergene ORFs (below, refer as intergenes), whose sequence lengths are ≥ 60 nt and genomic regions do not overlap with RNA-seq data in starvation and rich conditions [2], from the *S. cerevisiae* genome. Each extracted sequence also includes flanking regions which are 500 nt sequence long from both ends of an intergene ORF. While a large number of the intergene ORFs are expected to be non-genic, a small number of genic ones could still remain.

For each extracted sequence of the *de novo* protein-coding genes, we identified its orthologous sequences in *S. paradoxus*, *S. mikatae* and *S. bayanus* genomes. That is, we aligned it to each of the three genome sequences using glsearch [10], whose alignments are global in the query and local in the database sequences (global-local alignments), and collected sequences given by the best alignments for respective genomes. Since the flanking regions in an extracted sequence contain parts of protein-coding sequences of the neighboring genes in *S. cerevisiae* genome in most cases [11], this global-local alignment can be regarded as a synteny-based method. We regarded a set of extracted and collected sequences as orthologue.

A set of orthologous sequences was then aligned using MAFFT [12]. From the multiple sequence alignment, the most likely ancestral sequences before and after a gene was born *de novo* were constructed using FastML [13]. To obtain reliable ancestral sequences, we constructed ancestral sequences until the most recent common ancestor of *S. cerevisiae*, *S. paradoxus* and *S. mikatae*, because evolutionary distance between *S. cerevisiae* and *S. bayanus* is much greater than that between *S. cerevisiae* and *S. mikatae* [14]. Therefore, much of the analysis here was limited to ORF${}_{\textrm{S}_0}^+$ and ORF${}_{\textrm{S}_{1\sim 2}}$, where *S. mikatae* was used as an outgroup. Moreover, we discarded ancestral sequences whose $\frac{1}{L} \sum _{i=1}^L \max _{j \in \{\textrm{A,G,C,T}\}} p_{i,j}$ were less than 0.80, where *L* is the length of an ancestral sequence, and $p_{i,j}$ is the posterior probability of base *j* at position *i* in the sequence.

We focused here on the following statistical features observed in ancestral sequences before and after genes were born: (i) GC content, (ii) mutations accumulated, (iii) extension and shrinkage of ORF length, and (iv) appearance and disappearance of translation signature (Kozak consensus sequence). We evaluated these statistical features based on pairwise alignments of the ancestral sequences. Since aligned bases implies homologous bases, we can identify accumulated mutations, that is, aligned mismatch bases mean substitutions, and aligned bases and gaps mean indels. Moreover, we can also identify ancestral ORFs from recent ORFs by using consistency of reading frames and their overlapping length. As for (i), GC content of a genic ORF was calculated from base contents at the third codon positions of the ORF. Because base content at the third codon positions is mostly unconstrained by functional requirements, that is, by the need to code specific amino acids, the third codon position is a natural candidate for a predictive proxy of flanking GC content [15]. GC content of a non-genic ORF was calculated from base contents of entire region of the ORF. As for (ii), frequencies of substitutions and indels were calculated. Substitutions were classified into transition and transversion. Substitutions were also classified into ones concerned with GC pressure, that is, AT>CG and CG>AT, where $\alpha _1 \alpha _2 > \beta _1 \beta _2$ indicates that base $\alpha _1$ or $\alpha _2$ was substituted for base $\beta _1$ or $\beta _2$. As for (iii) and (iv), we defined a start codon of an ORF by the most upstream ATG and the most upstream Kozak consensus sequence (A..ATG and A..ATG.C) [16], where ‘.’ indicates any base.

## Results and discussion

### GC content

**Fig. 2:**
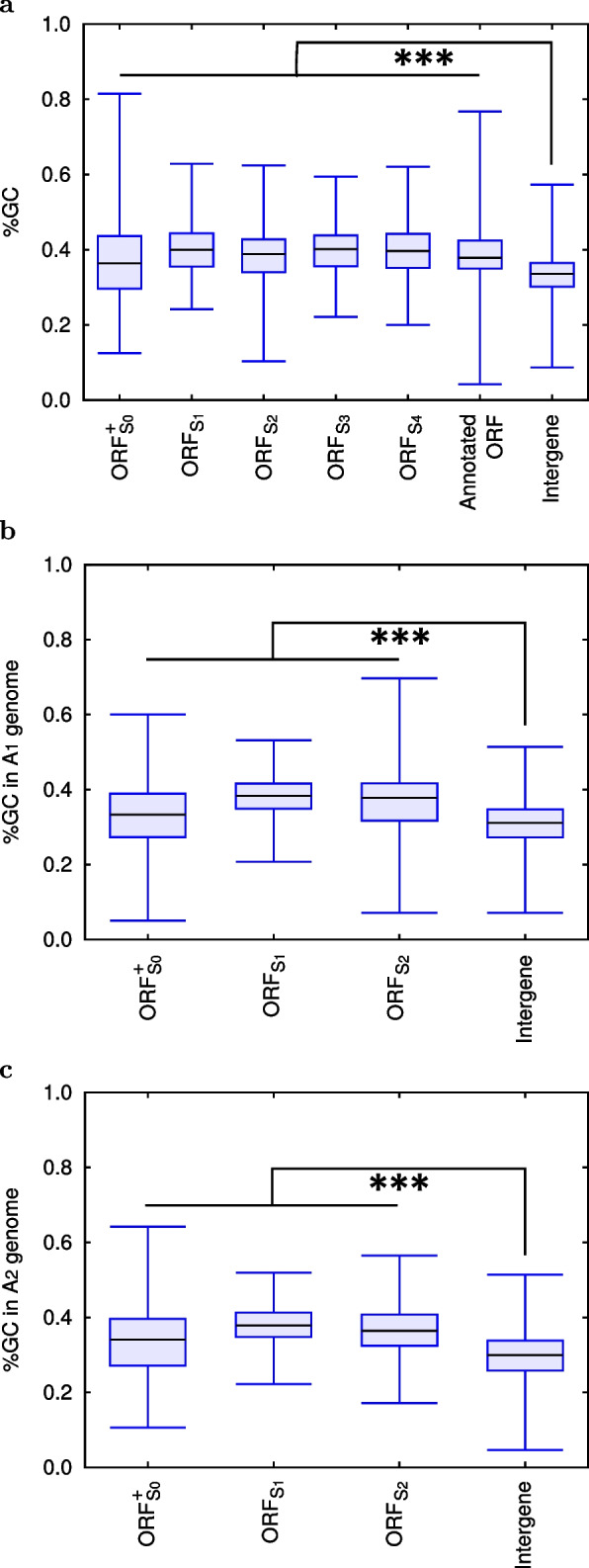
**a** A box plot representing GC contents (%GC) of *de novo* protein-coding genes (ORF${}_{\textrm{S}_0}^+$ and ORF${}_{\textrm{S}_{1\sim 4}}$), annotated ORFs and intergenes in the *S. cerevisiae* genome. %GC of *de novo* protein-coding genes are higher than that of intergenes and are comparable with that of annotated ORFs. Statistical differences between mean %GC of intergene and each of the others were evaluated by Welch’s *t*-test, and *p*-values were $\le 5.2 \times 10^{-14}$ (marked by ***). **b** A box plot representing %GC of *de novo* protein-coding genes (ORF${}_{\textrm{S}_0}^+$, ORF${}_{\textrm{S}_1}$ and ORF${}_{\textrm{S}_2}$) and intergene in the ancestral genome of A${}_1$ (see Fig. 1 for A${}_1$). %GC of *de novo* protein-coding genes are higher than that of intergene. Note that ORF${}_{\textrm{S}_0}^+$ and ORF${}_{\textrm{S}_1}$ at A${}_1$ are non-genic. Statistical differences between mean %GC of intergene and each of the others were evaluated by Welch’s *t*-test, and *p*-values were $\le 4.8 \times 10^{-6}$ (marked by ***). **c** A box plot representing %GC of *de novo* protein-coding genes (ORF${}_{\textrm{S}_0}^+$, ORF${}_{\textrm{S}_1}$ and ORF${}_{\textrm{S}_2}$) and intergene in the ancestral genome of A${}_2$ (see Fig. 1 for A${}_2$). %GC of *de novo* protein-coding genes are higher than that of intergene. Note that ORF${}_{\textrm{S}_0}^+$ and ORF${}_{\textrm{S}_{1\sim 2}}$ at A${}_2$ are non-genic. Statistical differences between mean %GC of intergene and each of the others were evaluated by Welch’s *t*-test, and *p*-values were $\le 1.1 \times 10^{-11}$ (marked by ***)

While *de novo* protein-coding genes in the catalogue are located in GC-rich regions of the *S. cerevisiae* genome (Fig. 2a), these regions had already been GC-rich before they were born (Fig. 2b and c). This indicates that *de novo* protein-coding genes are preferentially born from GC-rich non-genic regions. The similar preference was reported by Vakirlis et al. [17] using independent data sets of *S. sensu stricto*. Although it is known that long ORFs are more likely to be observed by chance in GC-rich regions, interestingly, they proposed the relationship to promoter activity intrinsic to such regions. See Table 2 for the detailed information of Fig. 2.

**Table 2:** **a** GC contents (%GC) of *de novo* protein-coding genes (ORF${}_{\textrm{S}_0}^+$ and ORF${}_{\textrm{S}_{1\sim 4}}$), annotated ORFs and intergenes in the *S. cerevisiae* genome. This table is the detailed information of Fig. 2a. **b** %GC of *de novo* protein-coding genes (ORF${}_{\textrm{S}_0}^+$, ORF${}_{\textrm{S}_1}$ and ORF${}_{\textrm{S}_2}$) and intergene in the ancestral genomes of A${}_1$ and A${}_2$ (see Fig. 1 for A${}_1$ and A${}_2$). This table is the detailed information of Fig. 2b and c

**a**				
Region	# of seq	%GC		
		Ave	SD	
ORF${}_{\textrm{S}_0}^+$	448	0.373	0.105	
ORF${}_{\textrm{S}_1}$	142	0.407	0.071	
ORF${}_{\textrm{S}_2}$	172	0.389	0.077	
ORF${}_{\textrm{S}_3}$	136	0.402	0.071	
ORF${}_{\textrm{S}_4}$	301	0.400	0.071	
Annotated ORFs	6,459	0.393	0.071	
Intergene	6,006	0.333	0.055	
**b**				
Region	Ancestral genome	# of seq	%GC	
			Ave	SD
ORF${}_{\textrm{S}_0}^+$	A${}_1$	274	0.333	0.084
	A${}_2$	259	0.337	0.089
ORF${}_{\textrm{S}_1}$	A${}_1$	115	0.383	0.055
	A${}_2$	112	0.381	0.055
ORF${}_{\textrm{S}_2}$	A${}_1$	144	0.377	0.089
	A${}_2$	142	0.368	0.071
Intergene	A${}_1$	5,283	0.309	0.055
	A${}_2$	5,304	0.299	0.063

### Mutations

**Fig. 3:**
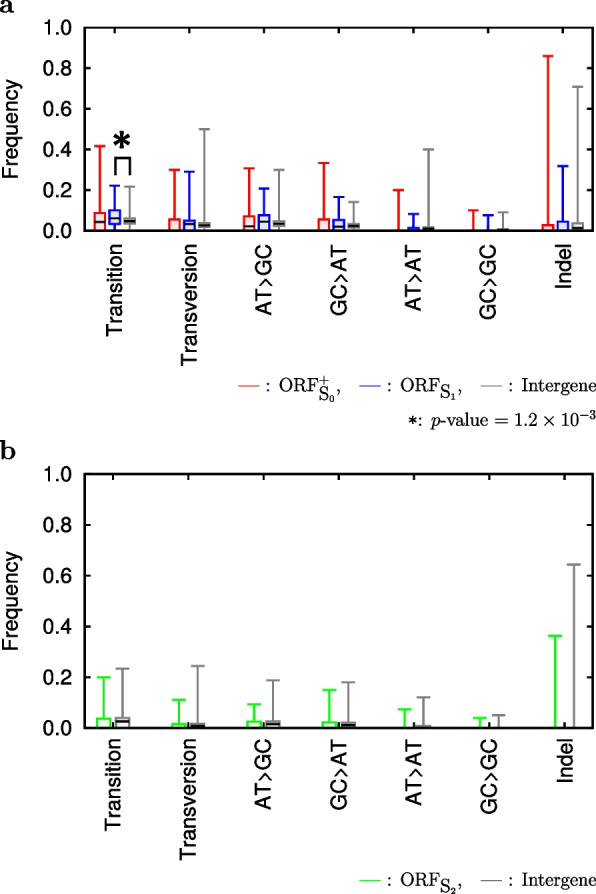
**a** A box plot representing frequencies of mutations (transition, transversion, …, and indel) accumulated in ORF${}_{\textrm{S}_0}^+$, ORF${}_{\textrm{S}_1}$ and intergene during the evolutionary time from A${}_1$ to *S. cerevisiae* (see Fig. 1 for A${}_1$). During this evolutionary time, ORF${}_{\textrm{S}_0}^+$ and ORF${}_{\textrm{S}_1}$ evolved from non-genic to genic sequences. Statistical differences between mean mutation frequency of intergene and each of the others were evaluated by Welch’s *t*-test, and significant difference was observed only in transition frequency between intergene and ORF${}_{\textrm{S}_1}$ (marked by *). **b** A box plot representing frequencies of mutations (transition, transversion, …, and indel) accumulated in ORF${}_{\textrm{S}_2}$ and intergene during the evolutionary time from A${}_2$ to A${}_1$ (see Fig. 1 for A${}_1$ and A${}_2$). During this evolutionary time, ORF${}_{\textrm{S}_2}$ evolved from non-genic to genic sequences. Statistical differences between mean mutation frequency of intergene and each of the others were evaluated by Welch’s *t*-test, and no significant differences were observed

Mutations that were accumulated during evolution of ORFs from non-genic to genic sequences were not significantly different from those that were accumulated in intergenes (Fig. 3), with few exceptions (* in Fig. 3a). Since mutations that are accumulated in intergenes are expected to be neutral, mutations that were accumulated during evolution of ORFs from non-genic to genic sequences are considered to be neutral. Lu et al. obtained similar results from independent data sets of *S. sensu stricto* [18]. However, there remains the probability that these mutations may contain a small amount of adaptive ones. See Table 3 for the detailed information of Fig. 3.

**Table 3:** **a** Frequencies of mutations (transition, transversion, …, and indel) accumulated in ORF${}_{\textrm{S}_0}^+$, ORF${}_{\textrm{S}_1}$ and intergene during the evolutionary time from A${}_1$ to *S. cerevisiae* (see Fig. 1 for A${}_1$). Average (Ave) and standard deviation (SD) of the frequencies are shown. During this evolutionary time, ORF${}_{\textrm{S}_0}^+$ and ORF${}_{\textrm{S}_1}$ evolved from non-genic to genic sequences. Numbers in parentheses are the numbers of sequences. This table is the detailed information of Fig. 3a. **b** Frequencies of mutations (transition, transversion, …, and indel) accumulated in ORF${}_{\textrm{S}_2}$ and intergene during the evolutionary time from A${}_2$ to A${}_1$ (see Fig. 1 for A${}_1$ and A${}_2$). Average (Ave) and standard deviation (SD) of the frequencies are shown. During this evolutionary time, ORF${}_{\textrm{S}_2}$ evolved from non-genic to genic sequences. Numbers in parentheses are the numbers of sequences. This table is the detailed information of Fig. 3b

**a**						
Mutations	A${}_1$ →*S.cer* (%)					
	ORF${}_{\textrm{S}_0}^+$		ORF${}_{\textrm{S}_1}$		Intergene	
	(697)		(116)		(5,569)	
	Ave	SD	Ave	SD	Ave	SD
Transition	5.35	6.50	6.63	5.16	4.89	2.26
Transversion	3.34	6.45	3.34	4.41	3.00	1.93
AT > GC	4.20	5.72	4.74	4.57	3.69	1.97
GC > AT	2.78	4.53	3.34	4.08	2.50	1.44
AT > AT	1.15	2.82	1.11	2.15	1.22	1.18
GC > GC	0.56	4.25	0.78	1.92	0.45	0.50
Indel	3.19	9.40	3.40	6.95	3.22	6.24
**b**						
Mutations	A${}_2$ →A${}_1$(%)					
	ORF${}_{\textrm{S}_2}$		Intergene			
	(143)		(5,490)			
	Ave	SD	Ave	SD		
Transition	2.35	3.96	2.43	2.24		
Transversion	1.01	2.00	1.06	1.34		
AT > GC	1.46	2.87	1.61	1.60		
GC > AT	1.22	2.29	1.25	1.33		
AT > AT	0.52	1.37	0.50	0.76		
GC > GC	0.16	0.62	0.13	0.29		
Indel	0.30	3.08	0.12	1.34		

### ORF length

**Fig. 4:**
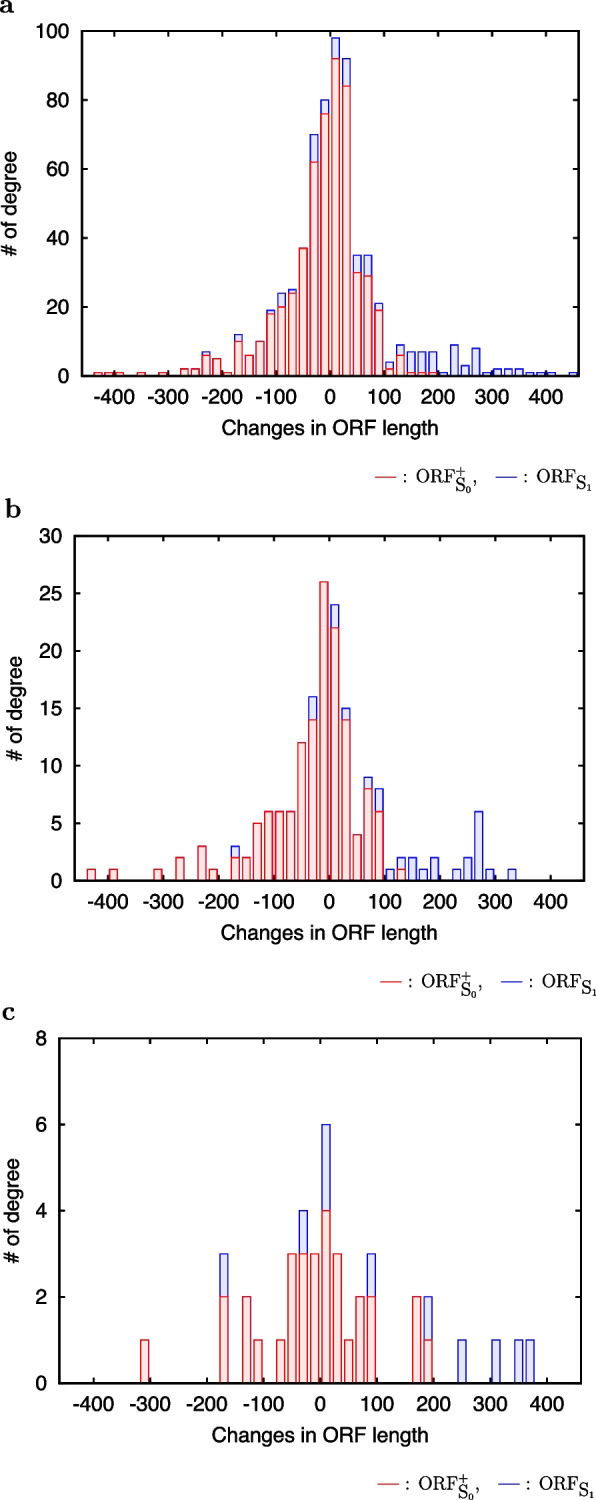
Changes in lengths of ORF$_{\textrm{S}_0}^+$ (red) and ORF$_{\textrm{S}_1}$ (blue) during the evolutionary time from A$_1$ to *S. cerevisiae* (see Fig. 1 for A$_1$). During this evolutionary time, ORF$_{\textrm{S}_0}^+$ and ORF$_{\textrm{S}_1}$ evolved from non-genic to genic sequences. **a** The most upstream ATG was used as a start codon of an ORF. **b** The most upstream A..ATG was used as a start codon of an ORF. **c** The most upstream A..ATG.C was used as a start codon of an ORF. These figures are the detailed information of Table 4

Although no significant trend in length changes of ORF${}_{\textrm{S}_0}^+$ was observed during the evolutionary time from A${}_1$ to *S. cerevisiae* (Table 4a), significant trend in length extension of ORF$_{\textrm{S}_1}$ was observed during this evolutionary time (Table 4b), where A$_1$ is the common ancestor of *S. cerevisiae* and *S. paradoxus* (Fig. 1). This difference is due to the difference in sequence length between ORF${}_{\textrm{S}_1}$ and ORF${}_{\textrm{S}_0}^+$. That is, the sequence length of ORF$_{\textrm{S}_1}$s, which are annotated ORFs, is long, and that of ORF${}_{\textrm{S}_0}^+$s, which are unannotated ORFs, is short, while ORF length of their orthologous regions in A$_1$ is short in both. Interestingly, while length changes of ORF${}_{\textrm{S}_0}^+$ are small, those of ORF${}_{\textrm{S}_1}$ are positively large (Fig. 4). These indicate that ORF${}_{\textrm{S}_0}^+$ was formed by repeating its extension and shrinkage by frameshift and nonsense mutations, and ORF${}_{\textrm{S}_1}$ were formed by combining other ORFs into itself by frameshift mutations.

**Table 4:** Changes in lengths of ORF$_{\textrm{S}_0}^+$ (**a**) and ORF${}_{\textrm{S}_1}$ (**b**) during the evolutionary time from A${}_1$ to *S. cerevisiae* (see Fig. 1 for A${}_1$). During this evolutionary time, ORF${}_{\textrm{S}_0}^+$ and ORF${}_{\textrm{S}_1}$ evolved from non-genic to genic sequences. Binomial test was used to detect significant trend in changes of ORF length. The background probabilities of the test were calculated from length changes in intergene ORFs during this evolutionary time (data not shown). Although no significant trend was observed for ORF${}_{\textrm{S}_0}^+$, significant trend was observed for ORF${}_{\textrm{S}_1}$ (marked by *** and **). When adopting the most upstream A..ATG.C as start codons, no significant trend was observed even for ORF${}_{\textrm{S}_1}$, because of small numbers of samples

**a**			
Changes in length	# of ORF${}_{\textrm{S}_0}^+$		
	The most upstream	The most upstream Kozak seq.	
	ATG	A..ATG	A..ATG.C
Extend	264	55	15
Shrink	285	88	16
No change	264	80	14
**b**			
Changes in length	# of ORF${}_{\textrm{S}_1}$		
	The most upstream	The most upstream Kozak seq.	
	ATG ${}^{\texttt {***}}$	A..ATG ${}^{\texttt {**}}$	A..ATG.C
Extend	83	24	8
Shrink	21	3	2
No change	10	4	0

$${}^\texttt {***}$$: $$P=1.3\times 10^{-9}$$, $$^\texttt {**}$$: $$P=9.8\times 10^{-5}$$

### Translation signature

Although ORF$_{\textrm{S}_0}^+$ and ORF$_{\textrm{S}_1}$ evolved from non-genic to genic sequences during the evolutionary time from A$_1$ to *S. cerevisiae* (see Fig. 1), no significant trend in appearance of Kozak sequences of them was observed during this evolutionary time (Table 5), where A$_1$ is the common ancestor of *S. cerevisiae* and *S. paradoxus* (Fig. 1). Carvunis et al., however, reported that proportion of *de novo* protein-coding genes with Kozak sequences increases as their conservation levels increase, that is, as they age [2]. Taken together, these two results indicate that there is a time delay between the appearance of Kozak sequences and the acquisition of protein-coding properties. This is in contrast to the simultaneous occurrence of the extension of ORF lengths and the acquisition of protein-coding properties. These may be due to the higher specificities of positions and bases of neutral mutations by which Kozak sequences appear compared to neutral mutations by which ORFs are extended. That is, the former mutations are ones to specific bases at specific positions surrounding start codons, while the latter mutations are ones with low base specificities in stop codons and overlapping regions of ORFs.

**Table 5:** Appearance and disappearance of Kozak sequences A..ATG (**a**) and A..ATG.C (**b**) during the evolutionary time from A$_1$ to *S. cerevisiae* (see Fig. 1 for A$_1$). During this evolutionary time, ORF$_{\textrm{S}_0}^+$ and ORF$_{\textrm{S}_1}$ evolved from non-genic to genic sequences. While binomial test was used to detect significant trend in appearance and disappearance of Kozak sequence, no significant trend was observed. The background probabilities of the test were calculated from those of intergene ORFs during this evolutionary time (data not shown)

**a**			
Kozak seq.	# of	# of	Total
A..ATG	ORF$_{\textrm{S}_0}^+$	ORF$_{\textrm{S}_1}$	
No	407	50	457
Retain	223	31	254
Disappear	176	51	227
Appear	58	20	78
**b**			
Kozak seq.	# of	# of	Total
A..ATG.C	ORF$_{\textrm{S}_0}^+$	ORF$_{\textrm{S}_1}$	
No	725	99	824
Retain	45	10	55
Disappear	32	8	40
Appear	27	9	36

### A putative scenario for *de novo* gene origination

To summarize all of the above, we found that (i) *de novo* protein-coding genes tend to originate from GC-rich regions, (ii) mutations that evolve ORFs from non-genic to genic sequences are neutral, and (iii) these mutations frequently extend/combine ORFs, (iv) followed by recruitment of translation signature (Kozak sequence). These findings naturally lead us to a putative a scenario of *de novo* origination of protein-coding genes. (1) In the beginning was GC-rich genome regions. (2) Neutral mutations were accumulated in the regions. (3) ORFs were extended/combined, and then (4) translation signature (Kozak sequence) was recruited. Interestingly, as the scenario progresses from (2) to (4), the specificity of neutral mutations increases (see previous subsection). That is, the order of events in the scenario is governed by ratio of number of neutral mutations being capable of causing each event to total number of all possible neutral mutations, and events occur in descending order of this ratio.

To the best of our knowledge, this is the first report outlining a scenario of *de novo* origination of protein-coding genes. As we mentioned the above, some of the statistical features in the scenario have been reported by independent researches [17, 18]. However, since each of them uses distinct data, there is insufficient evidence for outlining a single scenario from them. We applied here a systematic analysis to comprehensive data compiled by a single research group, then there is sufficient evidence for outlining a single scenario from statistical features observed. On the other hand, we should note that our scenario was derived from a *de novo* protein-coding gene catalogue containing ORF$_{\textrm{S}_0}^+$s which are free from overlap with annotated ORFs on the same strand [2]. Therefore, the ability of our scenario to explain process of *de novo* gene origination by overprinting and exonization [19] is limited.

### Advantages of our bioinformatic analysis

Our bioinformatic analysis has successfully sketched a putative scenario for *de novo* origination of protein-coding genes. The main advantage of our bioinformatic analysis is that we can directly observe the evolution from non-genic to genic sequences by constructing ancestral sequences. This direct observation enables our analysis to capture events that occur during a short evolutionary time. Therefore, the order of (3) and (4) in the above scenario was clearly shown by our analysis, while this order was not clear in existing analyses such as cross-species comparison [2]. As Klasberg et al. pointed out [20], the existing analyses can not fully capture fast evolution of *de novo* protein-coding genes. However, construction of ancestral sequences is fraught with ambiguity. Therefore, we adopted here conservative filters, such that we declined to construct the common ancestral sequences of *S. sensu stricto* (the 3rd paragraph of ‘Bioinformatic analysis’).

Another advantage of our analysis is that we can easily improve time resolution by incorporating closely related species data. *Saccharomyces boulardii* is located closer to *S. cerevisiae* than *S. paradoxus* on the *S. sensu stricto* phylogenetic tree [21]. By incorporating *S. boulardii* data, we can observe the evolution hfrom non-genic to genic sequences that occurs in a short period of time. On the other hand, *Saccharomyces kudriavzevii* is located where it fills the large gap between *S. mikatae* and *S. bayanus* on the *S. sensu stricto* phylogenetic tree [14]. By incorporating *S. kudriavzevii* data, we can observe the evolution from non-genic to genic sequences that occurred in older times.

### An insight into ‘ORF first’ model and ‘transcription first’ model

Finally, we provide an insight into ‘ORF first’ model and ‘transcription first’ model [22] from viewpoints of our research. For *de novo* origination of a protein-coding gene to occur, a non-genic sequence must both be transcribed and acquire an ORF before becoming translated. These events may in theory occur in either order, and there is evidence supporting both an ‘ORF first’ and a ‘transcription first’ model. On the other hand, our research suggested that events during *de novo* origination of protein-coding genes tend to occur in order of which they are likely to occur due to neutral mutations accumulated. This can lead us naturally to a conjectures below. In GC-rich regions, both of ‘ORF first’ and ‘transcription first’ might be observed, because they may already have relatively long ORFs and may already obtain weak transcriptional activity. In AT-rich regions, ‘ORF first’ might be mainly observed, because a few number of neutral mutations can extend ORF length, but cannot elevate GC content. Although they can also result in appearance of transcription factor binding sites, it is expected that its effect on *de novo* origination of protein-coding genes would be small compared to that of ORF extension.

## Data Availability

All data is available at http://labo.bio.kyutech.ac.jp/~ytlab/publication.html.

## Abbreviations

ORF: Open reading frame
A: Adenine
G: Guanine
C: Cytosine
T: Thymine
DNA: Deoxyribonucleic acid
RNA: Ribonucleic acid
URL: Uniform resource locator
